# Crystal structures of Bbp from *Staphylococcus aureus* reveal the ligand binding mechanism with Fibrinogen α

**DOI:** 10.1007/s13238-015-0205-x

**Published:** 2015-09-08

**Authors:** Xinyue Zhang, Meng Wu, Wei Zhuo, Jinke Gu, Sensen Zhang, Jingpeng Ge, Maojun Yang

**Affiliations:** Key Laboratory for Protein Sciences of Ministry of Education, Tsinghua-Peking Center for Life Sciences, School of Life Sciences, Tsinghua University, Beijing, 100084 China

**Keywords:** bone sialoprotein-binding protein (Bbp), fibrinogen, serine-aspartate repeat (Sdr), Microbial Surface Components Recognizing Adhesive Matrix Molecules (MSCRAMM), *Staphylococcus aureus*

## Abstract

Bone sialoprotein-binding protein (Bbp), a MSCRAMMs (Microbial Surface Components Recognizing Adhesive Matrix Molecules) family protein expressed on the surface of *Staphylococcus aureus (S. aureus)*, mediates adherence to fibrinogen α (Fg α), a component in the extracellular matrix of the host cell and is important for infection and pathogenesis. In this study, we solved the crystal structures of apo-Bbp^273−598^ and Bbp^273−598^-Fg α^561−575^ complex at a resolution of 2.03 Å and 1.45 Å, respectively. Apo-Bbp^273−598^ contained the ligand binding region N2 and N3 domains, both of which followed a DE variant IgG fold characterized by an additional D1 strand in N2 domain and D1′ and D2′ strands in N3 domain. The peptide mapped to the Fg α^561−575^ bond to Bbp^273−598^ on the open groove between the N2 and N3 domains. Strikingly, the disordered C-terminus in the apo-form reorganized into a highly-ordered loop and a β-strand G′′ covering the ligand upon ligand binding. Bbp^Ala298–Gly301^ in the N2 domain of the Bbp^273−598^-Fg α^561−575^ complex, which is a loop in the apo-form, formed a short α-helix to interact tightly with the peptide. In addition, Bbp^Ser547–Gln561^ in the N3 domain moved toward the binding groove to make contact directly with the peptide, while Bbp^Asp338–Gly355^ and Bbp^Thr365–Tyr387^ in N2 domain shifted their configurations to stabilize the reorganized C-terminus mainly through strong hydrogen bonds. Altogether, our results revealed the molecular basis for Bbp-ligand interaction and advanced our understanding of *S. aureus* infection process.

## INTRODUCTION

*Staphylococcus aureus (S. aureus)* has been one of the leading causes of bacterial infections worldwide. Each year, some 500,000 patients in United States’ hospitals contract staphylococcal infections. *S. aureus* resides as part of the normal flora in the healthy human body until there is damage to skin surface or mucosal barrier, when it can gain access to tissues or the bloodstream, ultimately resulting in a wide range of infections and diseases, including impetigo, bacteremia, endocarditis, sepsis and arthritis (Lowy, 1998). Several antibiotics have been introduced to successfully treat *S. aureus* infections in patients over the past few decades. However, the infections became a growing concern lately due to the emergence of highly virulent and antibiotic-resistant strains, leading to increased morbidity and mortality (Zetola et al., 2005). Effective vaccines against *S. aureus* at early stages of infection are highly desirable, although all efforts to develop these vaccines have failed to date (Deresinski and Herrera, 2010; Vazquez et al., 2011).

*S. aureus* has evolved multiple strategies to promote colonization and evade the immune system. The initial adhesion of the pathogen to the extracellular matrix (ECM) components of the host is believed to be a critical step for successful infection. This is mediated by *S. aureus* surface adhesins called Microbial Surface Components Recognizing Adhesive Matrix Molecules (MSCRAMMs) (Gillaspy et al., 1998; Patti et al., 1994). Several structurally related proteins characterized by serine-aspartate dipeptide repeats (SD repeats) make up a family of MSCRAMMs (McCrea et al., 2000). The serine-aspartate repeat (Sdr) family include SdrF and SdrG in *S. epidermidis* and clumping factor A (ClfA), ClfB, SdrC, SdrD, SdrE and Bbp in *S. aureus* (Josefsson et al., 1998; McDevitt et al., 1994; Ni Eidhin et al., 1998; Tung et al., 2000). *S. aureus* bone sialoprotein-binding protein (Bbp) is an allelic variant of SdrE (Peacock et al., 2002). The members of Sdr family are predicted to adopt a similar structural pattern (Trad et al., 2004). A secretary signal sequence locates at the N-terminus followed by a ligand-binding A region and a characterized R region composed of SD repeats. The C-terminus features a cell wall-anchoring motif including the conserved LPXTG sequence (W), a hydrophobic membrane-spanning domain (M) and a short positively charged cytoplasmic tail (C) (Downer, 2002). In addition, SdrC, SdrD, SdrE and Bbp have different numbers of B repeats inserted between region A and R with the presence of a well-defined 12 residues cation-binding EF-hand loop (Josefsson et al., 1998). Our recent work showed that B1 domain interacted with N2 domain and opened the ligand binding cleft between N2 and N3 domains in SdrD (Wang et al., 2013).

The gene identified from chromosomal DNA isolated from *S. aureus subsp. aureusTCH60* encodes bone sialoprotein-binding protein (Bbp) with 1149 amino acids, containing SD-repeats of 154 residues and the ligand-binding A region from 53 to 601 residues further divided into N1, N2 and N3 domains. *S. aureus* isolated from patients suffering from septic arthritis and osteomyelitis specifically interacts with bone sialoprotein (BSP), a noncollagenous protein of bone and dentine extracellular matrix, mediated by Bbp (Ganss et al., 1999; Ryden et al., 1987; Tung et al., 2000). BSP is proposed to induce hydroxyapatite crystal formation and distributes predominantly in the newly formed bone, which is more likely to be infected (Hultenby, 1994; Hunter and Goldberg, 1993).

Fibrinogen (Fg), a hexameric glycoprotein consisting of three different chains α_2_, β_2_ and γ_2_, plays critical roles in blood clotting and thrombosis (Gailit et al., 1997; Kollman et al., 2009; Mosesson et al., 2001). ClfB binds to fibrinogen α (Fg α) chain (Ganesh et al., 2011; Xiang et al., 2012). ClfA and the fibronectin-binding proteins FnbpA and FnbpB all bind to the C-terminal residues of fibrinogen γ (Fg γ) chain (Rivera et al., 2007; Wann et al., 2000). SdrG is reported to attack the thrombin cleavage site of fibrinogen β (Fg β) chain (Davis et al., 2001). A “dock, lock and latch” (DLL) model is identified in SdrG-Fg β complex to elucidate the ligand binding mechanism, where the ligand docks in the opened groove between N2 and N3 domains and the C-terminus across the groove stretches into N2 domain (Ponnuraj et al., 2003).

As a bifunctional MSCRAMM, Bbp also recognizes the human Fg α chain and inhibits thrombin induced blood coagulation (Vazquez et al., 2011). The molecular basis for Bbp-ligand interaction remains unknown. In our study, we solved the crystal structures of apo-Bbp^273−598^ and Bbp^273−598^ complexed with the peptide of Fg α^561−575^. We described the N2-N3 domains of Bbp similar to Dev-IgG fold (Deivanayagam et al., 2002). The Bbp^273−598^-Fg α^561−575^ complex revealed the ligand-binding basis through the rearrangement of the C-terminus and the significant changes in four regions. These results advance our understanding of the ligand binding mechanism of Bbp during *S. aureus* infection. Moreover, our study should shed light on the further identification of the substrate or ligand of other closely related Sdr proteins, and provide novel targets for the development of potent antagonists, vaccines or antibiotics.

## RESULTS

### Structure of apo-Bbp^273−598^

**Figure 1:**
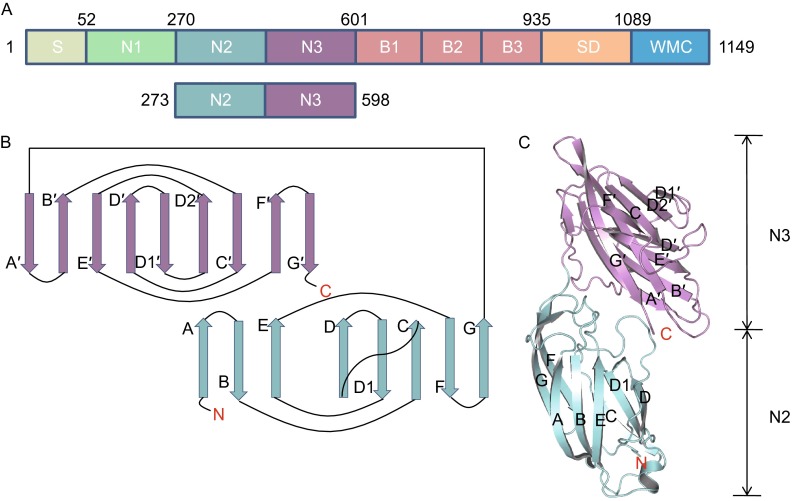
**Overall structure of the apo-Bbp** ^**273−598**^. (A) Domain organization of the Bbp molecule. S (amino acid 1–52), signal sequence; N1–N3 (amino acid 53–601), Fg binding region; B (amino acid 602–935), B-repeats region; R (amino acid 936–1089), serine-aspartate repeat region; W, wall-spanning domain; M, membrane anchor; C, cytoplasmic positively charged tail. The region of N2 and N3 domains for crystallization (below). (B) Schematic representation of the topology of Bbp^273−598^ fold. The N2 and N3 domains are shown in cyan and violet, respectively. The N- and C-terminus are marked with red characters. (C) Cartoon representation of apo-Bbp^273−598^ structure with its N- and C-terminus indicated. The N2 and N3 domains are shown in cyan and violet, respectively

It was previously reported that on other MSCRAMMs of the Sdr family, the ligand-binding region was mapped to the N2 and N3 domains of the N-terminal A region. Based on sequence alignment of Bbp, SdrG and ClfA (Davis et al., 2001; Josefsson et al., 1998), we engineered a plasmid that would generate a recombinant fusion protein covering residues Asn^273^–Pro^598^ of Bbp from *S. aureus*, a segment containing both N2 and N3 domains (Fig. 1A), with N-terminal GST-tag for purification purposes in *Escherichia coli*. We obtained apo-Bbp^273−598^ protein and solved the structure at 2.03 Å resolution (Table 1), consisting of residues Asn^273^–Leu^584^ and additional residues Gly and Ser at N-terminus, two of the remaining five amino acid residues (GPLGS) from digested GST-tag (Fig. 1C). No electron density was observed for the 14 residues at C-terminus in the apo-Bbp^273−598^ structure.

**Table 1:** Statistics of data collection and refinement

	Peptide free	Fg α bound
Data collection		
Space group	*I222*	*P2*_*1*_
*a*, *b*, *c* (Å)	96.241,98.924, 102.257	60.916, 74.961, 75.563
*α*, *β*, *γ* (°)	90.00, 90.00, 90.00	90.00, 102.91, 90.00
Wavelength (Å)	0.979	0.979
Resolution (Å)	2.03 (2.10–2.03)	1.45 (1.50–1.45)
*R*_merge_ (%)	7.8 (44.8)	6.3 (75.5)
*I*/*σ*	15.6 (2.9)	26.8 (1.6)
Completeness (%)	96.8 (89.3)	99.2 (95.8)
Redundancy	3.7 (3.5)	5.1 (4.5)
Wilson B factor (Å^2^)	29.7	17.7
Refinement		
*R*_factor_	21.42	17.68
*R*_free_	26.41	21.09
No. atoms	2467 protein atoms + 172 solvent atoms + 2 Ca^2+^	5125 protein atoms + 1 Mg^2+^ + 159 peptide atoms + 1026 solvent atoms
B factors		
Overall	38.794	21.274
RMSD bond lengths	0.008	0.006
RMSD bond angles	1.177	1.081
Ramachandran plot statistics (%)		
In favored regions	96.5	98.9
In allowed regions	2.9	1.1
In disallowed regions	0.6	0.0

Values in parentheses are for the highest resolution shell. *R* = Σ|*F*_*obs*_ − F_*calc*_|/Σ_*Fobs*_, where F_*calc*_ is the calculated protein structure factor from the atomic model (*R*_free_ was calculated with 5% of the reflections selected)

The apo-Bbp^273−598^ folds into two distinct domains N2 and N3, both of which have two layers of β-sheets and are structurally similar to the Dev-IgG fold (Fig. 1B), a variant of IgG fold (Deivanayagam et al., 2002). The two β-sheets of the N2 domain are composed of A, B and E strands on one side and C, D, D1, F and G strands on the opposite side. In N3 domain, C′, D1′, D2′, F′ and G′ strands form one principal sheet and A′, B′, D′ and E′ strands contribute to the facing sheet. The additional D1′ and D2′ strands present the featured Dev-IgG fold. One difference occurs with regard to the D strand in N2 domain, which parallels with E strand, although exhibiting an antiparallel orientation with the corresponding strand in the description of SdrG, ClfB and ClfA (Ganesh et al., 2008; Ponnuraj et al., 2003; Xiang et al., 2012).

In the apo-Bbp^273−598^ structure, the C-terminus with poor electron density extends into the solvent region, thus leading to an open groove. Presumably, a ligand-binding site could exist in the groove. (All structural figures in this paper were generated by PyMOL).

### Structure of the Bbp^273−598^-Fibrinogen α (Fg α)^561−575^ complex

**Figure 2:**
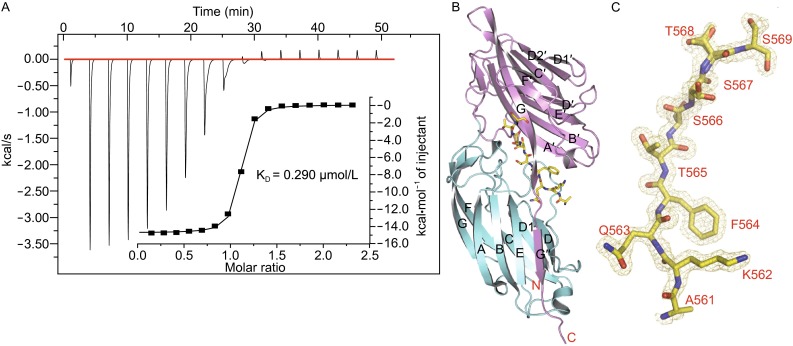
**Overall structure of the Bbp** ^**273−598**^ **-Fg α** ^**561−575**^ **complex**. (A) ITC curves of Fg α^561−575^ titrated into Bbp^273−598^ protein. The first peak in the thermogram has not been used for analysis. (B) Cartoon representation of Bbp^273−598^-Fg α^561−575^ complex structure with its N- and C-terminus indicated. The N2 and N3 domains are colored the same as in Fig. 1C. The peptide is shown as sticks in yellow. (C) 2Fo-Fc map of Fg α^561−575^ peptide. The map is contoured at the level of 1.0 δ. The peptide residues are marked with red characters

The ITC result showed that Fg α^561−575^, a synthesized polypeptide Fg α-chain containing residues 561–575 (Vazquez et al., 2011), has binding affinity to Bbp^273−598^ with a *K*_D_ of 0.290 μmol/L (Fig. 2A). To study the molecular mechanism underlying Bbp-ligand recognition, Bbp^273−598^ was crystallized in complex with the ligand Fg α^561−575^. We solved the crystal structure of Bbp^273−598^-Fg α^561−575^ complex at 1.45 Å (Fig. 2B and Table 1).

Each crystallographic asymmetric unit contains two independent Bbp^273−598^-Fg α^561−575^ molecules. The bound peptide Fg α^561−575^ threads into the groove between the N2 and N3 domains in a snug conformation, with a well-defined 2Fo-Fc electron density map observed for the residues Lys^562^–Ser^569^ of the N-terminus of the peptide (Fig. 2C). The six residues of the C-terminus of Fg α^561−575^ have few interactions with the ligand binding groove and are not traceable in the density map. Due to the poor side-chain density, the N-terminal residue Fg α^Ser561^ was replaced with an Alanine during structure refinement.

**Figure 3:**
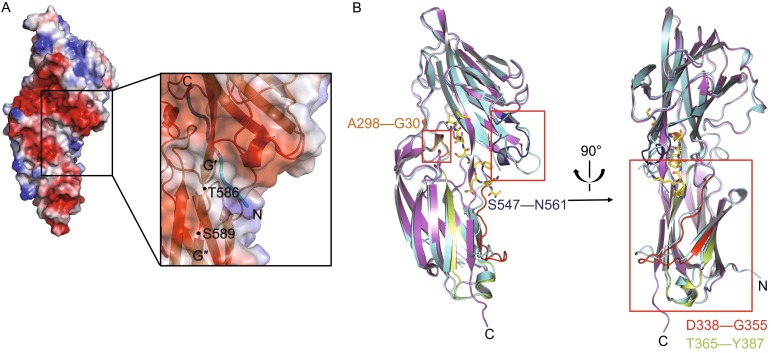
**Conformational changes occur to C-terminus and additional four regions**. (A) Surface charge representation of Bbp^273−598^ binding with the Fg α^561−575^ peptide. The surface is colored depending on negative charge, electrically neutral area and positive charge that are visualized in red, white and blue, respectively. Close view of surface and cartoon representation of C-terminus of Bbp^273−598^ and the Fg α^561−575^ peptide shown as ribbon representation colored in cyan. The connecting loop is shown as cartoon representation, composed of the residues Thr586–Ser589 running across the open groove are marked. (B) Structure alignment of apo-Bbp^273−598^ and Bbp^273−598^-Fg α^561−575^ shows four changed regions. Apo-Bbp^273−598^ and Bbp^273−598^-Fg α^561−575^ are colored in cyan and magenta, respectively. The peptide is shown as sticks colored in yellow. The regions Bbp^Asp338–Gly355^, Bbp^Thr365–Tyr387^, Bbp^Ala298–Gly301^ and Bbp^Ser547–Gln561^ of the Bbp^273−598^-Fg α^561−575^ complex are show in red, lemon, orange and blue, respectively

Structure comparison of Bbp^273−598^ between the apo-protein and in complex with the Fg α^561−575^ shows that the RMSD for the Cα atoms is 0.679 Å. Even though the overall topology of Bbp^273−598^ is similar in the two structures, significant conformational changes were observed around the peptide-binding groove, including the C-terminus of Bbp^273−598^ and four additional regions containing Bbp^Asp338–Gly355^, Bbp^Thr365–Tyr387^, Bbp^Ala298–Gly301^ in N2 domain and Bbp^Ser547–Gln561^ in N3 domain, respectively (Fig. 3).

### The structural basis for peptide binding

**Figure 4:**
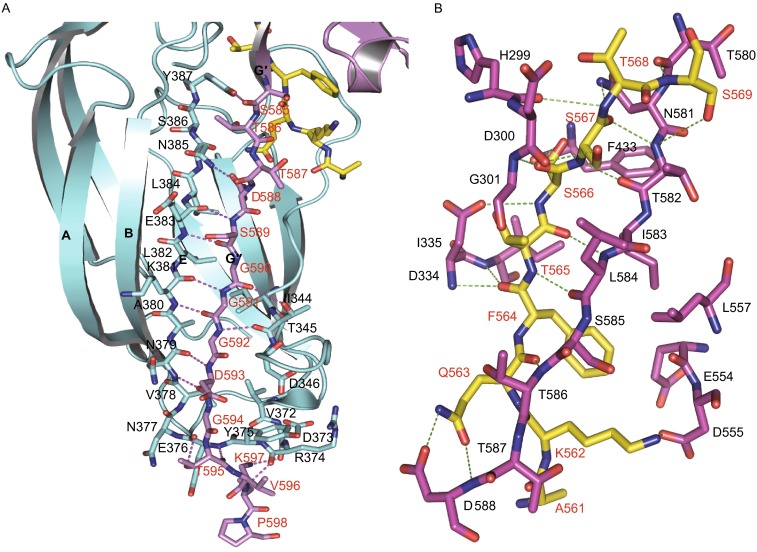
**Detailed interactions in the four changed regions**. (A) Closer view of the interactions between the C-terminus of N3 and the regions Bbp^Asp338–Gly355^ and Bbp^Thr365–Tyr387^ of N2 in Bbp^273−598^-Fg α^561−575^; the residues involved are shown as sticks and marked with red and black characters, respectively. The hydrogen bonds are indicated by magenta dashed lines. The peptide is shown as sticks in yellow. (B) Detailed interactions between the ligand and Bbp^273−598^ in the Bbp^273−598^-Fg α^561−575^ complex. Bbp^273−598^ and Fg α^561−575^ are shown as sticks, colored in magenta and yellow, respectively. The residues of Bbp^273−598^ and Fg α^561−575^ are marked with black and red characters, respectively. The hydrogen bonds are indicated by green dashed lines

The disordered C-terminus encompassing residues Ser^585^–Pro^598^ in apo-Bbp^273−598^ rearranges in the Bbp^273−598^-Fg α^561−575^ structure. The connecting loop between the G′ and G′′ strands spanning residues Thr^586^-Gly^589^ runs across the central region of the groove and the following sequence forms a short β-strand G′′ which inserts into N2 domain (Fig. 3A), making regions Bbp^Asp338–Gly355^ and Bbp^Thr365–Tyr387^ deviate significantly from the apo-form (Fig. 3B). It shows a deviation of 5.6 Å for Bbp^Pro347^ in the region Bbp^Asp338–Gly355^ between the C and D strands in N2 domain. Bbp^Gly590 and Gly592^ of β-strand G′′ of N3 domain form two hydrogen bonds with Bbp^Thr345^ in this region. The region Bbp^Thr365–Tyr387^ in N2 domain contains β-strand E and the TYKFTDYVD sequence, a TYTFTDYVD-like motif conserved in Sdr protein family (McCrea et al., 2000). The β-strand E moves toward β-strand G′′ to stabilize the C-terminus of N3 through several hydrogen bonds. Bbp^Tyr387^ in this region interacts with Bbp^Ser585^ at the end of the G′ strand, which play an important role in redirecting the C-terminus. Bbp^Asp373 and Arg374^ in this region form two hydrogen bonds with Bbp^Lys597^ to stabilize the tail of the reordered C-terminus (Fig. 4A).

In addition, the binding of the peptide also induces a reorganization of the region Bbp^Ala298–Gly301^ between the A and B strands in N2 domain and a large movement of the region Bbp^Ser547–Gln561^ between the E′ and F′ strands in N3 domain toward the peptide binding groove. Two newly formed α-helices are observed in both of the two regions (Fig. 3B). The residues Bbp^His299 and Gly301^ in the first α-helix interact with Fg α^Thr568 and Ser566^ through two main-chain hydrogen bonds and the Bbp^Asp300^ forms the third hydrogen bond with the side-chain of Fg α^Ser567^. In the second α-helix, Bbp^Asp555^ contributes a hydrogen bond with Fg α^Lys562^ and the side chain of Bbp^Leu557^ contacts with the aromatic ring of Fg α^Phe564^ mediated by a hydrophobic interaction (Fig. 4B).

The structural rearrangements and the direct protein-ligand interactions formed upon peptide binding result in an effectively stabilized Bbp-Fg α complex compared to its apo-form.

### Structural insights into Bbp^273−598^ and Fg α^561−575^ interactions

Apart from the interactions between the Fg α^561−575^ peptide and the residues from the two newly formed α-helices we have described above, there are several contacts with distances of less than 4 Å marked (Fig. 4B). Among them, there are three pairs of antiparallel main-chain hydrogen bonds between residues Fg α^Ser567, Thr565 and Gln563^ and Bbp^Thr582, Leu584 and Thr586^ in the G′ strand and the connecting loop region. The carbonyl group of Fg α^Ser567^ interacts with the side-chain polar group of Bbp^Asn581^ forming another hydrogen bond. The residue Fg α^Ser569^ from the C-terminus of the peptide forms a hydrogen bond with Bbp^Thr580^ in the G′ strand. The side chain hydroxyl group of Fg α^Ser566^ interacts with the backbone atom of Bbp^Phe433^ in N3 domain and Fg α^Ser566^ forms the second hydrogen bond with the side-chain carbonyl group of Bbp^Asp334^ in N2 domain. Fg α^Phe564^ contributes two hydrogen bonds with Bbp^Asp334 and Ile335^ in the loop region between the C and D strands in N2 domain. The backbone atom of Bbp^Asn581^ interacts with the carbonyl group of Fg α^Ser569^, which plays a significant role in anchoring the C-terminus of the Fg α^561−575^ peptide. Two hydrogen bonds formed between the polar group of Fg α^Gln563^ and Bbp^Asp588^ from the loop region are involved in locking the peptide N-terminus.

### Analysis of the interactions between Bbp^273−598^ mutants and Fg α^561−575^

**Figure 5:**
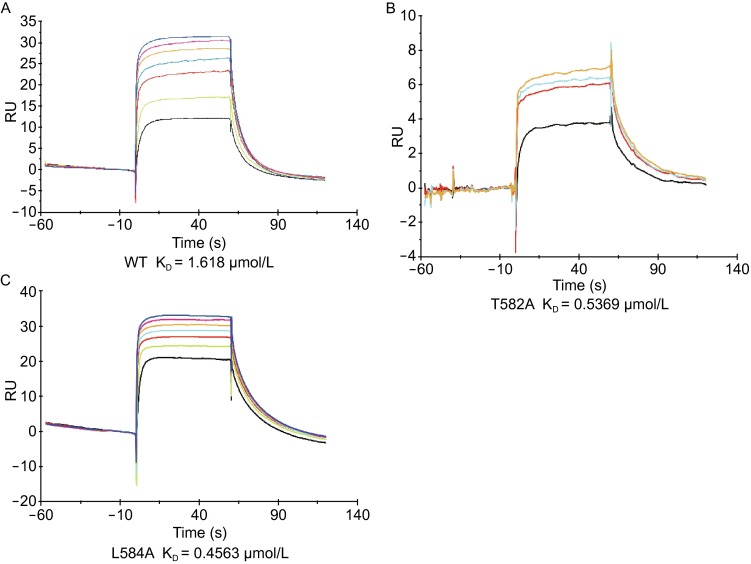
**SPR analyses of the interactions between Bbp** ^**273−598**^ **WT and mutants (T582A and L584A) and the Fg α** ^**561−575**^ **(A–C), respectively**. The proteins were immobilized on a BIA-core sensor chip. The concentration of Fg α^561−575^ peptide ranges from 62.5 μmol/L to 0.976 μmol/L: Night blue, 62.5 μmol/L; Magenta, 31.25 μmol/L; Peach, 15.625 μmol/L; Bright blue, 7.812 μmol/L; Red, 3.9 μmol/L; Lime, 1.953 μmol/L; Black, 0.976 μmol/L. K_D_ values of individual binding assays are indicated below the sensorgrams

Mutagenesis studies were conducted to further verify the binding of Fg α^561−575^ to Bbp^273−598^. We mutated the residues Thr^582^ and Leu^584^ to Ala respectively which form two pairs of hydrogen bonds with the peptide. The mutant proteins were purified to homogeneity and tested for their interaction with the Fg α^561−575^ peptide by surface plasmon resonance (SPR) (Fig. 5). The results indicate that the mutated protein Bbp^T582A^ or Bbp^L584A^ exhibits higher binding affinities with the peptide than wild type (WT) Bbp^273−598^ protein. This is probably because the side chain of Ala occupies less space compared to Thr or Leu, which brings an alteration of steric hindrance. Thus, the alteration presumably makes the peptide more easily dock into the groove and contacts more tightly with Bbp^273−598^. Even though Bbp^Thr582 and Leu584^ could interact with the peptide by two pairs of main chain-main chain hydrogen bonds (Fig. 3C), the replacement to alanine might serve a similar role instead of breaking the interaction with the peptide according to the results here. Perhaps, we can speculate that the residues Bbp^Thr582 and Leu584^ are only involved in the binding with the peptide, but not showing specificities on ligand recognition.

## DISCUSSION

In this study, we have solved the crystal structures of apo-Bbp^273−598^ and the Bbp^273−598^-Fg α^561−575^ complex. Based on the structural information, we analyzed the structural basis for ligand binding.

**Figure 6:**
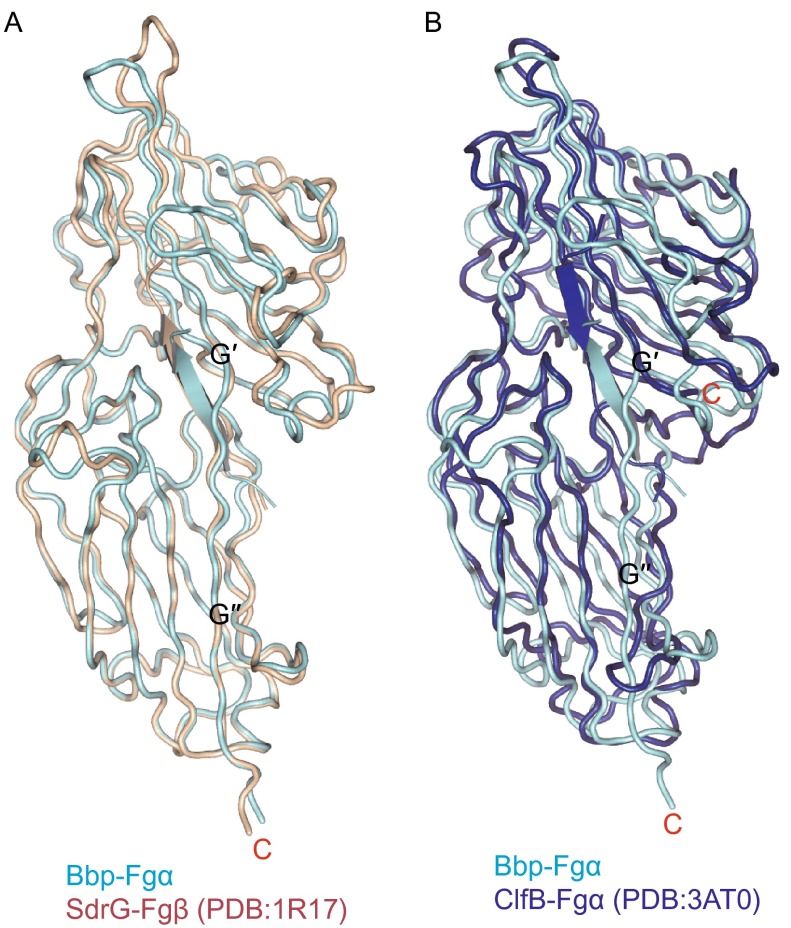
**Structure comparison of Bbp, ClfB and SdrG**. (A) Superimposition of Bbp-Fg α and SdrG-Fg β (PDB entry: 1R17), colored in cyan and wheat, respectively. The peptides in both structures are shown as cartoon. The C-termini of Bbp and SdrG are indicated. (B) Superimposition of Bbp-Fg α and ClfB-Fg α (PDB entry: 3AT0), colored in cyan and blue, respectively. The peptides in both structures are shown as cartoon. The C-termini of Bbp and ClfB are indicated

In our study, tight interactions between the protein and the ligand result in a stable binding state. Due to the “Dock” of the ligand, the rearrangements occur to C-terminus and additional four regions of Bbp^273−598^. The connecting loop covers the open groove, resulting in “Lock” of the ligand. And then the G′′ strand forms compact interactions with the E strand in the region Bbp^Thr365–Tyr387^ of N2 domain, which “Latch” the ligand binding site and thus stabilize the overall structure. Our structure further supports the DLL model described for the SdrG-Fg β complex (Fig. 6A) (Ponnuraj et al., 2003).

In the work of V. Ganesh et al. on the ClfB-ligand complex, the ligand binding mechanism was described as the “DL” model due to the absence of the “Latch” process (Ganesh et al., 2011). In their structure, peptide Fg α^336−347^ in ClfB adopts a reverse orientation compared to the peptide Fg α^561−575^ in our structure. The C-terminus of ClfB does not stretch into N2 domain to interact with the E strand but exhibits a different direction. Upon ligand binding, no rearrangements are observed in the region between the D and D′ strands in N2 and the region between the E and F strands in N3 (Fig. 6B). However, large movements occur to the corresponding regions Bbp^Asp338–Gly355^ in N2 and Bbp^Ser547–Gln561^ in N3 of Bbp^273−598^ in our studies. The diversity of the ligands binding pattern of MSCRAMMs adds to the necessity for structural analysis of individual members of this family.

Altogether, our findings have promoted the understanding of the ligand binding mechanism of Bbp in Sdr family, a critical step in the *S. aureus* infection process. In addition, potential new target sites based on these pathogen-host interactions could be explored for development of potent antibiotics and new therapeutic methods.

## MATERIALS AND METHODS

### Cloning, expression and purification of the recombinant proteins

The fragment of the Bbp gene (corresponding to 273–598 aa) was amplified using *S. aureus* ATCC 25923 genomic DNA by PCR. The gene fragments of mutated proteins Bbp^T582A^ and Bbp^L584A^ followed the same protocol. After digestion with *Bam*HI and *Xho*I (NEB), the amplified fragments were cloned into the prokaryotic expression vector pGEX-6p-1 (GE Healthcare Life Sciences) to produce the GST-Bbp fusion protein and were confirmed by DNA sequencing. The recombinant protein was expressed in *Escherichia coli* strain BL21 with a high yield.

The bacteria cells were harvested and resuspended in lysis buffer containing 1× PBS, 2 mmol/L DTT and 1 mmol/L PMSF. The cells were homogenized by sonification and cell debris was removed completely by centrifuging at 13,000 rpm for 50 min at 4°C.

The recombinant protein was firstly purified by GST-affinity column and digested with PreScission protease overnight. The eluant was collected and concentrated for further purification using gel filtration chromatography (Superdex-200 column, GE Healthcare) in buffer containing 20 mmol/L HEPES pH 7.5, 200 mmol/L NaCl, 2 mmol/L DTT on the FPLC system (GE Healthcare Life Sciences). The proteins in every step of purification were analyzed by SDS-PAGE.

### Crystallization and structure determination

The apo-Bbp^273−598^ and its complex were concentrated to 30 mg/mL in 20 mmol/L HEPES pH 7.5, 200 mmol/L NaCl, 2 mmol/L DTT. Crystals were screened by the sitting-drop vapor diffusion method (Jancarik et al., 1991) using sparse-matrix screen kits Crystal Screen I and II (Hampton Research), followed by optimizing the crystallization conditions through the variation of protein concentrations and pH. Crystals were grown at 18°C using the hanging-drop vapor diffusion method by mixing 1.0 μL protein solution with 1.0 μL reservoir solution and equilibrating against 200 μL reservoir solution. The apo-Bbp^273−598^ crystals were grown in 0.2 mol/L calcium acetate hydrate, 0.1 mol/L sodium cacodylate trihydrate pH 6.5, 18% PEG8000. The synthesized Fg α^561−575^ peptide was added into the concentrated protein samples at 10:1 ratio and the protein-peptide complex crystals are grown in 0.2 mol/L lithium sulfate, 0.1 mol/L Tris-HCl pH 8.2, 30% PEG4000. The apo-Bbp^273−598^ and Bbp^273−598^-peptide complex crystals diffracted to 2.03 Å and 1.45 Å respectively. The data were collected at the Shanghai Synchrotron Radiation Facility (SSRF) BL17U using a MAR225 (MAR Research, Hamburg) CCD detector at 100 K and processed with the HKL2000 package (Otwinowski and Minor, 1997). Further processing was carried out using programs from the CCP4 suite (Collaborative Computational Project, 1994). The model building of the Bbp^273−598^ molecules was conducted in COOT and the structure with peptide was determined by molecular replacement methods in CCP4 (Emsley and Cowtan, 2004). All the structures were refined with the PHENIX packages (Adams et al., 2002). Data collection and structure statistic are summarized in Table 1.

### Synthesis of Fg α^561−575^ chain peptide

The peptide corresponding to the fibrinogen α^561−575^ was synthesized as previously described (Vazquez et al., 2011).

### Isothermal titration calorimetry

ITC experiments were carried out at 25°C using a Microcal iTC200 instrument (GE Healthcare). The cell contained 50 μmol/L Bbp^273−598^ and the syringe contained 500 μmol/L peptide in the buffer containing 200 mmol/L NaCl and 20 mmol/L HEPES pH 7.5. Injecting peptide into buffer was performed as the blank titration. The data were fitted and analyzed using the Origin 7 software package (Microcal).

### Surface plasmon resonance spectroscopy

The interaction affinities between Fg α^561−575^ and Bbp^273−598^ protein were conducted by surface plasmon resonance (SPR) using BIAcore T200 instrument (GE Healthcare). The wild type Bbp^273−598^ protein and two mutants were immobilized, respectively, on a CM5 sensor chip in 10 mmol/L sodium acetate, pH 4.5. The immobilization level was 3375RU. The synthetic peptide in 200 mmol/L NaCl and 20 mmol/L HEPES (pH 7.5) was injected on the protein-coated surface at various concentrations at 30 μL/min. The binding and dissociation was 60 s and 120 s, respectively.

## ABBREVIATIONS

Bbp: bone sialoprotein-binding protein
BSP: bone sialoprotein
Clf: clumping factor
ECM: extracellular matrix
Fg: fibrinogen
MSCRAMM: Microbial Surface Components Recognizing Adhesive Matrix Molecules
RMSD: root mean square deviation
*S. aureus*: *Staphylococcus aureus*
Sdr: serine-aspartate repeat
SPR: surface plasmon resonance
WT: wild type
